# Structural Basis for the Lipopolysaccharide Export Activity of the Bacterial Lipopolysaccharide Transport System

**DOI:** 10.3390/ijms19092680

**Published:** 2018-09-10

**Authors:** Greg Hicks, Zongchao Jia

**Affiliations:** Department of Biomedical and Molecular Sciences, Queen’s University, Kingston, ON K7L 3N6, Canada; 8gah@queensu.ca

**Keywords:** lipopolysaccharides, membrane transport, Gram-negative bacteria

## Abstract

Gram-negative bacteria have a dense outer membrane (OM) coating of lipopolysaccharides, which is essential to their survival. This coating is assembled by the LPS (lipopolysaccharide) transport (Lpt) system, a coordinated seven-subunit protein complex that spans the cellular envelope. LPS transport is driven by an ATPase-dependent mechanism dubbed the “PEZ” model, whereby a continuous stream of LPS molecules is pushed from subunit to subunit. This review explores recent structural and functional findings that have elucidated the subunit-scale mechanisms of LPS transport, including the novel ABC-like mechanism of the LptB_2_FG subcomplex and the lateral insertion of LPS into the OM by LptD/E. New questions are also raised about the functional significance of LptA oligomerization and LptC. The tightly regulated interactions between these connected subcomplexes suggest a pathway that can react dynamically to membrane stress and may prove to be a valuable target for new antibiotic therapies for Gram-negative pathogens.

## 1. Introduction

Gram-negative bacteria protect themselves from chemical stressors by incorporating hydrophobic lipopolysaccharide (LPS) into their outer membranes. The LPS coating acts as a physical barrier to antibiotics and has antigenic properties that make it critical to understanding and countering pathogenicity. Individual LPS are amphipathic, consisting of a hydrophobic lipid moiety (Lipid A) and a hydrophilic oligosaccharide core (OS). Some bacteria add an O-antigen (Oag), a repeating saccharide moiety that extends out from the OS core. The LPS coating is critical to the integrity of the cellular envelope, and Gram-negative bacteria need to replenish it at an astonishing rate to survive.

LPS is synthesized at the cytoplasmic side of the inner membrane (IM) before it is transported to the outer membrane (OM). Lipid A is synthesized in the cytoplasm via the Raetz pathway and then ligated to the OS core [1]. It is then flipped to the outer leaflet by the ATP-binding cassette (ABC)-type transporter MsbA [2]. The O-antigen is synthesized and transported to the periplasm by a parallel pathway before it is ligated to the OS [3,4]. Producing LPS in the cytoplasm presents the physiologically daunting task of getting the amphipathic LPS across two membranes, the periplasm and a substantial concentration gradient. Gram-negative bacteria accomplish this through the specialized transportation machinery of the LPS transport (Lpt) system, a linear protein bridge that spans the cellular envelope.

**Figure 1:**
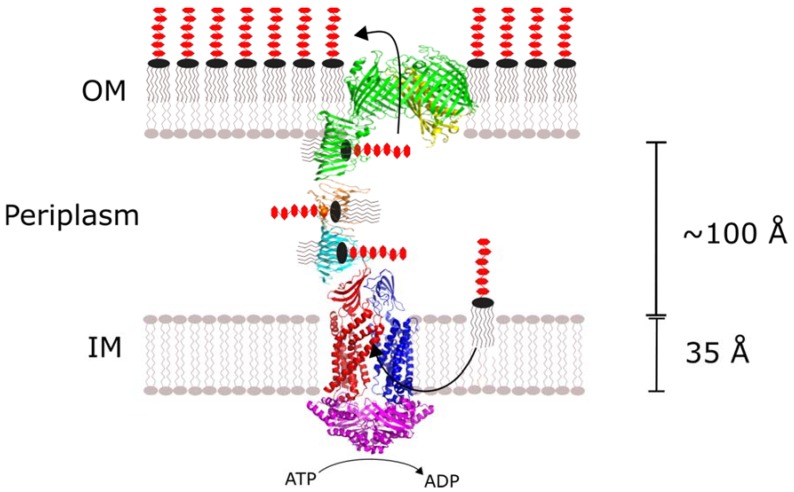
The Lpt (LPS transport) system forms a continuous protein bridge across the inner membrane, periplasm and outer membrane. LptB (purple), LptG (red) and LptF (blue) extract LPS (lipopolysaccharide) from the inner leaflet of the IM (inner membrane) through an ATPase-dependent mechanism. LPS cross the periplasm along a continuous hydrophobic groove formed by LptC (cyan), LptA (orange) and LptD (green). LptD and LptE (yellow) sort LPS to the outer leaflet of the outer membrane (OM).

The Lpt system is an oligomeric complex consisting of Lpt proteins A through G. The membrane-bound LptB, F, G and C subunits are connected to the LptD/E heterodimer in the OM by periplasmic LptA (Figure 1). The LptB_2_FG tetramer extracts LPS from the outer leaflet of the IM and provides the energy to drive LPS transport through an ATPase-dependent mechanism. LptC and LptA provide a continuous LPS binding surface that conveys it to the OM. There, LptD/E inserts LPS laterally into the outer leaflet of the OM where it is dispersed across the extracellular surface. With some species-dependent exceptions, all seven of the Lpt proteins are essential to LPS transport and Gram-negative bacteria survival.

The LPS coating of the Gram-negative outer membrane is a literal, physical barrier to the development of new antibiotics. Needing to cross the lipid barrier limits the physiochemical properties of antibiotic compounds to narrow ranges of size and lipophilicity [5]. Disrupting the Lpt system can increase cell permeability, and its OM-bound components make it more immediately accessible. The Lpt system is therefore an excellent target for new antibiotics to directly target cell survival or complement other compounds. Further characterization of the Lpt system’s subunit-to-subunit interactions and LPS bindings could contribute to the rational design of new antibiotics for the treatment of Gram-negative pathogens.

Until recently, there were major gaps in the experimentally-derived structures of the Lpt subunits. The periplasmic LPS-binding domain of LptD was absent from the available structure, making it difficult to visualize how the Lipid A and OS moieties crossed the membrane together. On the IM side of the complex, the lack of LptF and LptG structures limited the structural characterization of LptB’s extraction mechanism to predictions based on other ABC transporter systems. With the publication of the complete LptD/E and LptB_2_FG structures solved by Botos et al. and Luo et al., respectively, a complete picture of the Lpt system’s components is available [6,7]. These structures have answered longstanding questions about the mechanisms of LPS transport and prompted intriguing new ones about its assembly and regulation.

The recent breakthroughs in Lpt structure were accompanied by validation of the protein-bridge “PEZ” model of LPS transport. The model wherein the Lpt subunits form a continuous complex from the IM to the OM and LPS is propelled along it continuously by the ATPase activity of LptB has long been favoured over the periplasmic dissolution or membrane-junction models. It is consistent with the individual interactions between the Lpt subunits and the structural features of the Gram-negative cellular envelope, but direct evidence of the complete bridge and its activity was not observed until a recent study where Sherman et al. demonstrated the Lpt-dependent transmission of LPS between proteoliposomes [8]. Between their findings and the newly-solved Lpt structures, a more complete structural-functional model of LPS transport is now possible. 

This review was prompted by the crossing of these two major milestones in Lpt research. The essentiality of the Lpt system and its specific role in preserving the impermeability of the outer membrane make it a promising target for antibiotics, and these new findings could lead to the development of new rationally-designed inhibitors. Questions remain as to how the specific interactions within the Lpt complex facilitate LPS binding and transport. This review will summarize the current research on the structural interactions within the Lpt system and discuss their potential implications regarding function and regulation of OM biogenesis overall.

## 2. The LptB_2_FG Complex Drives LPS Extraction from the IM to the Periplasm

**Figure 2:**
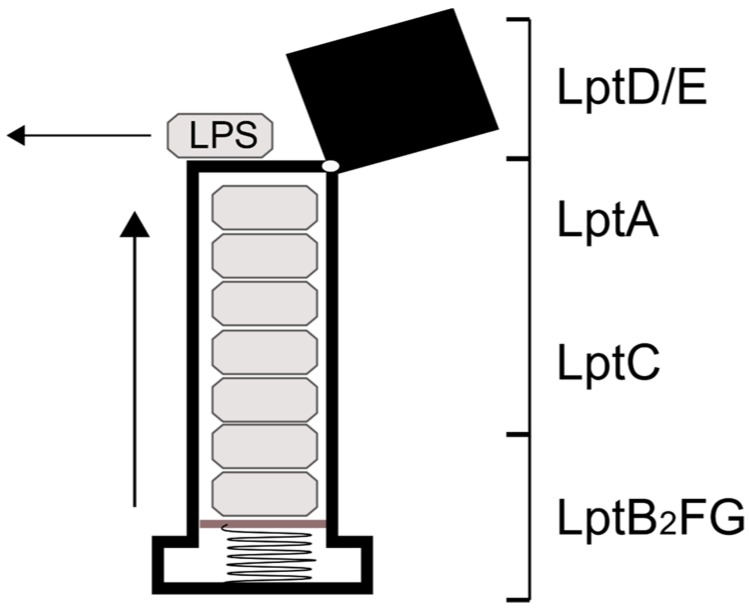
The mechanism of LPS transport to the outer membrane is often referred to as the “PEZ” model in reference to the spring-loaded candy dispenser. In this analogy, the LptB_2_FG complex is the “spring” that pushes the LPS “candy” through the chamber (LptA and LptC) to the cap (LptD/E), which is lifted to release them one at a time.

The overall mechanism of the Lpt system is often likened to a “PEZ” candy dispenser (Figure 2), wherein a spring at the bottom of a candy-filled chamber slides the contents up as candies are removed from the top one at a time. In vivo, this means that every LPS-binding site in the assembly is continuously passing the molecule from the previous subunit to the next until it reaches the OM. Obviously, this description does not account for how the individual Lpt subunits bind and release LPS, but it is useful for emphasizing the core role of the LptB_2_FG complex as the “spring” that provides the motivating force for the LPS “candy”. 

The subcomplex of LptF, LptG and dimeric LptB spans the IM and is responsible for extracting LPS from the membrane and propelling it along the periplasmic bridge. The LptB dimer is located in the cytoplasm, where it binds and hydrolyzes ATP. LptB forms a complex with the transmembrane LptF/G dimer. This configuration resembles other ABC transporters: a cytoplasmic nucleotide-binding domain (NBD) hydrolyzes ATP to switch the substrate cavity of the transmembrane domains (TMD) between inward- and outward-facing conformations [9]. In this case, the LptB dimer hydrolyzes ATP to alter the conformation of the transmembrane LptF/G dimer. However, the mechanism of the LptB_2_FG complex necessarily differs from other ABC transporters because it extracts its substrate directly from the same leaflet of the membrane, rather than switching it from one side of the membrane to another. LptB also differs from other ABC NBDs because it retains some ATPase activity even when purified in vitro, whereas other ABCs are inactive without the full complex [10]. The complete LptB_2_FG complex displays substantially higher ATPase activity, but curiously, activity decreases by 40% with the inclusion of LptC [7]. Until recently, these differences were difficult to explore as much of LptB_2_FG’s structural mechanism had to be inferred from other ABC complexes. 

LptB’s catalytic activity couples to the LptF/G heterodimer’s extraction of LPS like other ABC transporters, wherein the coupling helices of the TMD interact with the variable Q-loop of the NBD. Structural comparison of ATP-and ADP-bound LptB shows that ATP binding, hydrolysis and release induce conformational changes in the Q-loop region, mediated predominantly by two conserved residues (F90 and R91) [11]. The interaction between LptB and LptF/G evidently has some activating effect on the catalytic domain, as ATPase activity is substantially higher with the full complex. LptB_2_FG activity and subsequent LPS transport are further enhanced in the presence of the antibiotic novobiocin, a hydrophobic DNA gyrase inhibitor [12]. Novobiocin binds to the Q-loop and directly interacts with F90 and R91, strongly indicating that LptF/G’s effect on LptB ATPase activity is mediated directly through their coupling interaction. The actual mechanism of this effect remains unknown, but novobiocin is a potentially promising lead compound for the rational design of antibiotics that target LPS export from the IM.

**Figure 3:**
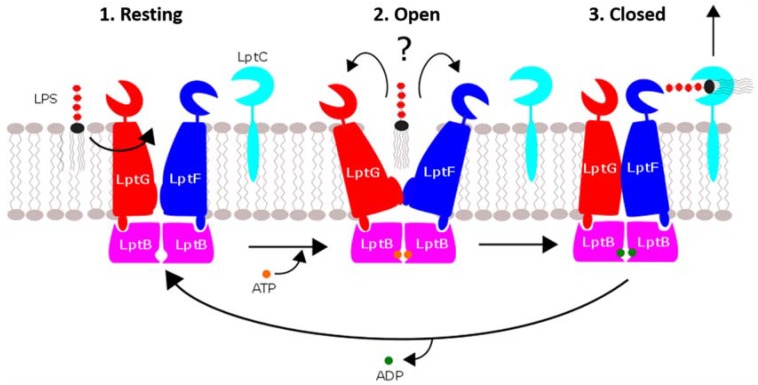
The mechanism of LPS transport to the outer membrane is often referred to as the “PEZ” model in reference to the spring-loaded candy dispenser. In this analogy, the LptB_2_FG complex is the “spring” that pushes the LPS “candy” through the chamber (LptA and LptC) to the cap (LptD/E), which is lifted to release them one at a time.

The crystal structures of the LptB_2_FG tetramer solved by Luo et al. indicate a mechanism whereby the complex cycles through three conformational states to extract LPS from the IM to the periplasm [7] (Figure 3):

1. Resting: The LptB nucleotide-binding sites are unoccupied, and the LptF/G cavity is oriented inwards.
2. Open: ATP binds LptB, inducing the LptF/G cavity to open away from the IM, and receives the Lipid A moiety of LPS, which is still embedded in the IM.
3. Close: LptB hydrolyzes ATP, inducing the LptF/G cavity to close again. LPS is forced out of the IM into the periplasm.

Based on this mechanism, Luo et al. have suggested that LptB_2_FG represents a third distinct type of ABC transporter, deemed type-III. 

The overall mechanism of LptB_2_FG is consistent with structural observations, but LPS’s immediate destination following extraction from the IM remains ambiguous. The hydrophobic grooves of the LPS-binding domains of LptF and LptG are oriented away from and towards the IM, respectively, suggesting they may have differing functions in the transmission of LPS. Luo et al. theorized that the two may alternate as the binding site for LPS, although LPS binding to either component has yet to be reported [7]. Induced crosslinking experiments have successfully characterized specific LPS interactions with LptA and LptC, so a similar approach or other in vitro experiments could further elucidate the proposed mechanism. Simpson et al. hypothesized that LptF/G either extracts LPS directly through their LPS-binding domains or triggers the handoff indirectly by stimulating a conformational change in LptC [13]. Now that a general overview of the conformational changes that LptB_2_FG undergoes is available, further investigation of how LPS interacts with its various domains is needed to understand how it facilitates transport to the other Lpt subunits.

How the LptB_2_FG complex interacts with the other IM-bound subunit, LptC, and how that influences LPS extraction are still open questions. LptC reduces the ATPase of the activity of the complex in vitro, even though its localization to the periplasmic side of the IM would imply no direct interaction with LptB [7]. Although functional studies have confirmed that LptC is the intermediate between the IM complex and LptA, how or why it influences LptB’s activity remains unclear.

## 3. LptC’s Role in IM-Periplasm Transport Is Ambiguous

LptC is essential to LPS transport and cell survival, but the function of several of its structural elements is unclear. LptC consists of a C-terminal periplasmic domain that closely resembles the other LPS-binding domains and a single N-terminal transmembrane helix. Its periplasmic domain complexes with LptB_2_FG and LptA and has been shown to bind LPS in vivo. Although LptC is bound to the IM, the N-terminal helix is not essential to Lpt assembly, as completely truncating it does not affect cell survival or the formation of the LptB_2_FG complex [14]. LptC may localize to the IM to improve the efficiency of Lpt assembly, but its specific interactions with the other subunits are sufficient for LPS transport.

LptC’s essentiality is dependent on specific interactions with LptF. Benedet et al. found that cells lacking LptC could be made viable by mutating a single LptF residue (R212) [15]. Membrane permeability increases as a result, but some LPS is still able to reach the surface. The likeliest explanation is that the IM complex is able to recruit an additional LptA to receive LPS in the absence of LptC. This conditional dependence also suggests that wildtype LptF forms a specific interaction with LptC that normally excludes LptA. Benedet et al. proposed that LptC could be a late evolutionary addition to what used to be a six-subunit complex [15]. Martorana et al. also showed that overexpressing LptB compensates for truncation of LptC’s C-terminus (residues 139–191), possibly by shifting the binding equilibrium in favor of the full complex [16]. If this is the case, LptC may be important to the efficient and stable assembly of the LptB_2_FG complex, in addition to directly transporting LPS.

LptC’s periplasmic domain might serve a role in stabilizing the LptB_2_FG complex, boosting the efficiency of the Lpt system. Although it has been theorized that the truncated LptC may be able to form an alternative edgewise interaction with LptA, this seems less likely than the alternative suggested by Benedet et al. wherein LptA is able to receive LPS from the IM independent of LptC [15]. The C-term LptC mutation could reduce the stability of the overall LptB_2_FGC complex, so increased LptB expression could compensate by shifting the binding equilibrium in favor of the LptB2FG complex.

LptC has also been shown to form a homodimer, but no functional significance has been identified. The N-terminal edges of LptC’s periplasmic domains form a head-to-head dimer in vitro [17]. Mutations disrupting this interaction have little effect on LptC’s ability to bind LPS or LptA [18]. LptC’s ability to dimerize may just be a side-effect of the β-jellyroll fold’s modularity, but it may be worth accounting for in future investigations of its in vivo function.

## 4. LPS-Binding Domains Span the Periplasm

LptA and the periplasmic domains of LptC, LptD, LptF and LptG have divergent sequences, but share a common OstA-like twisted β-jellyroll fold that binds the Lipid A moiety of LPS. The 16 antiparallel β-strands form a pair of β-sheets in a “V” shape. The interior of the “V” forms a hydrophobic groove along the interior that widens slightly near the N and C-termini [19]. This groove likely shields Lipid A from the periplasmic environment, while the hydrophilic OS core and O-antigen remain exposed. The β-jellyroll domains oligomerize from head-to-tail with a 90° twist per subunit, forming a continuous multiprotein β-sheet and a spiraling hydrophobic groove. This rod-like oligomer provides the path for LPS from the IM to OM. The relative arrangement of the β-jellyroll domains is well-established, and residues essential to oligomerization have been identified; however, how these interactions contribute to LPS transport beyond providing the shielding Lipid A in the periplasm is still under investigation. 

The Lpt β-jellyroll folds are very similar to one another, but the positioning of their N-terminal helices varies. LptA, LptD and LptG have short N-terminal helices that loop around to the C-terminal end of the hydrophobic groove of the jellyroll [6,7,19]. LptC and LptF lack similar helices: the N-terminus of LptC is disordered, and the N-terminal end of the LptF periplasmic domain links directly to helix 5 of its TM domain [7,20]. In LptD, the N-terminal helix forms one of two disulfide bonds that are essential to functional assembly of the LptD/E complex.

The apo structures of LptA, C and D indicate that the β-jellyroll must undergo extensive conformational changes to receive and subsequently hand off LPS. The hydrophobic cavity observed in the crystal structures of the empty LPS-binding domains are too small to accommodate the fatty acid chains of Lipid A [6,7,19]. A recent series of electron paramagnetic resonance (EPR) experiments by Schultz et al. demonstrated that LPS binding induces a substantial rearrangement in the inward-facing side of the hydrophobic groove of LptA and LptC [17,21]. The largest change is a partial unfolding of the N-terminal edge of the pocket, while the C-terminal edge remains relatively immobile. 

It was previously thought that the transfer of LPS from the periplasmic domain of LptC to LptA is driven in part by the latter’s higher affinity for LPS. In the absence of ATP, LptA has been shown to displace LPS from LptC in solution [20]. However, more recent assessments of the LPS affinity of LptC and LptA suggest the difference may be small or even inverted, with *K_d_* values ranging from 11–28 and 7–35 uM, respectively [17,21]. The transmission of LPS between Lpt β-jellyroll subunits may therefore be a more dynamic process than the “PEZ” model implies alone, whereby the periplasmic bridge undergoes “ripples” of ordered-disorder transitions that shuffle LPS along its length. 

## 5. LptA Oligomerization and Membrane Stress

**Figure 4:**
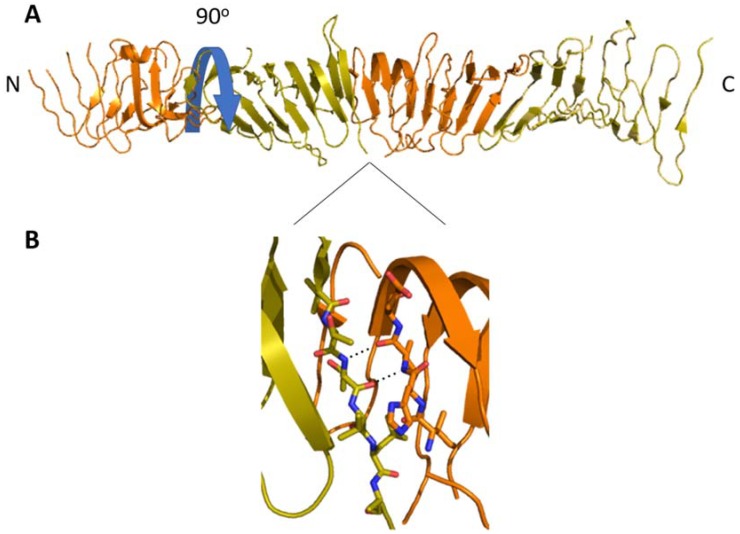
(**A**) The β-jellyroll domains of LptA form a rod-like head-to-tail multimer (alternating orange and gold) in solution with an ~90° rotation per subunit (blue arrow). (**B**) The N- and C-terminal β-strands form a continuous β-sheet along the length of the multimer. This is likely analogous to LptA‘s interactions with the β-jellyrolls of LptC and LptD.

LptA’s function and relative placement in the Lpt system is understood, but its stoichiometry in vivo remains uncertain. LptA is the only component of the Lpt system that lacks a transmembrane component, as it spans the periplasmic space between the IM and OM complexes. LptA co-purifies almost exclusively with membrane fractions when expressed at native levels, indicating that most LptA is bound to other Lpt components rather than floating freely in the periplasm [22]. The first crystal structure of LptA solved by Suits et al. showed it arranged in an end-to-end fibrous tetramer, which forms a continuous hydrophobic groove between the LptA monomers (Figure 4). Mass spectral analysis later confirmed that LptA forms 2–5-member oligomers in a concentration-dependent manner when purified in vitro and that the resultant complexes are stabilized by LPS [23]. UV-dependent dimers of modified LptA have been purified, although these could be experimental artifacts resulting from the presence of tags or altered expression [24]. 

The LptA-LptA binding interface probably uses the same contacts as LptA-LptC and LptA-LptD, so it is difficult to investigate the in vivo relevance of LptA oligomerization via mutagenesis without also disrupting LPS transport. The recent development of high-resolution in situ imaging techniques like electron cytomography may circumvent this issue by making it possible to directly measure the transenvelope length of the Lpt complex, although it may be necessary to first identify conditions that favour LptA oligomerization [25].

It is tempting to speculate that LptA oligomerization allows the Lpt system to tolerate changes in the intermembrane space resulting from osmotic stress. A single-LptA complex can bridge a typical *Escherichia coli* intermembrane distance of 100 Å, but the width of the periplasm could vary between species and in response to osmotic stress. In *E. coli*, the LptA appears to express at the same level as the other monomeric components of the Lpt system under normal conditions. However, functional analysis of the promoters associated with LptA shows additional responsiveness to changes in LPS biogenesis [26]. It is possible that the Lpt complex varies between single and multiple-LptA states in response to increased LptA availability, influenced either by the disruption of a single-subunit complex or independent increases in expression.

LPS’s last stop in the periplasm is the β-jellyroll domain of LptD. The interaction between LptA and LptD appears to be a key regulatory checkpoint in the assembly of the functional Lpt complex. LptA binds LptD/E preferentially to LptC, so during Lpt assembly, it is likely recruited to the OM before connecting to the IM subcomplex [22]. LptA binds the β-jellyroll of LptD in a head-to-tail interaction like the other periplasmic domains and forms no direct interactions with LptE [27]. However, LptA does not bind LptD if its specific disulfide bonds are not formed correctly [24]. It is intriguing that such a small difference would disrupt the otherwise robust β-jellyroll oligomerization process, especially given that the LptD subunit shows a great deal of rotational flexibility relative to the OM [6]. This requirement may be a specific adaptation that prevents malformed LptD from connecting with the rest of the Lpt system. 

Recent structural advancements in our understanding of the Lpt complex suggests an ability to dynamically respond to changes to cellular envelopes’ dimensions. The Rcs stress response system in enterobacteria was recently shown to respond to changes in periplasmic width, suggesting that essential transenvelope complexes are adapted to transenvelope stress [28]. Given how crucial the Lpt system is to cell survival, it stands to reason that it would be adapted to compensate for expansions and contractions of the periplasmic space resulting from osmotic stress. LptA could allow the system to tolerate changes in intermembrane distance by recruiting newly-detached membrane translocons. 

## 6. LptD/E Inserts LPS into the Outer Membrane Laterally

**Figure 5:**
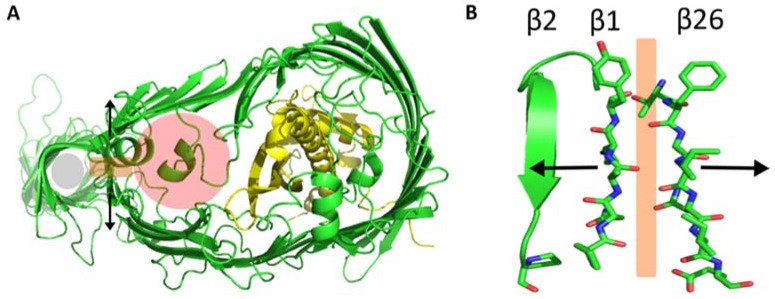
(**A**) The top-down view of the crystal structure of LptD (green) and LptE (gold) shows how the Lipid A and OS moieties of LPS exit the IM through openings in the N-terminal β-jellyroll (grey) and β-barrel domain (pink) respectively. This is made possible by the lateral gate that forms between the two domains. (**B**) Strands β1 and β26 of the transmembrane β-barrel separate perpendicular to the path of LPS. Proline residues on β1 and β2 (P231 and P246) limit the strands’ ability to form a sheet, which allows β1 and β2 to separate.

Until recently, the available structures of the LptD/E dimer were insufficient to fully confirm how the OM complex conveys the Lipid A and OS moieties to the outer leaflet. The C-terminal TM portion of LptD is a massive 26-strand β-barrel, and the LptE lipoprotein sits inside its lumen. The full-length structure solved by Botos et al. shows that the N-terminal domain of LptD is a β-jellyroll that extends from the periplasm and into the hydrophilic core of the OM [6]. This suggests a two-portal mechanism whereby Lipid A is inserted directly into the membrane by the β-jellyroll, while the OS component proceeds through the hydrophilic lumen of the β-barrel (Figure 5).

A lateral gate in the β-barrel of LptD allows the Lipid A and OS moieties to proceed to the outer leaflet of the OM. Conserved phenylalanine residues opposite one another on the β1 and β2 strands of the barrel prevent β1 from forming a complete hydrogen bonding network with β26 [6]. This allows β1 and β26 to slide apart, so the OS and O-antigen moieties can move out of the barrel to the cell surface. Functional LptD/E’s very specific disulfide bond configuration restricts the size of the lateral gate on the periplasmic side, possibly to prevent incorrect insertion of LPS into the inner leaflet of the OM. The extracellular side of the barrel lumen is rich in negatively-charged residues, which likely help to repel the similarly charged saccharide moieties out of the complex. LptD’s two-portal structure gives LPS a clear path from the periplasm to its final destination on the extracellular surface.

## 7. LptE Is a Multifunction Facilitator of LPS Export

Unlike the other Lpt proteins, LptE’s essential functions are not obvious from the overview of the “PEZ” model. LptE is embedded into the LptD lumen, but it is inserted into the OM independently by the Lol pathway [29]. LptE is a lipoprotein with little structural similarity to the other Lpt components, consisting of two α-helices closely associated with a four-strand β-sheet [30]. Rather than act as a direct LPS transporter, LptE appears to have three have overlapping functions in LPS transport: functional LptD assembly, preserving membrane impermeability and distributing LPS to the cell surface.

Functional assembly of LptD requires LptE as a template. LptD assembly requires the assistance of the BAM complex and extensive reshuffling of disulfide bonds [31,32]. Without the correct disulfide bond configuration, LptD cannot bind LptA or export LPS. LptD is still inserted into the OM when LptE is depleted, but the population of functionally-oxidized protein is depleted, as well. A three-residue LptE mutant (PIS117–119R) induces a similar effect [33]. The affected region of LptE may act as a template for LptD that ensures the cysteine residues are correctly positioned for disulfide bond rearrangement.

LptE also preserves membrane impermeability by plugging LptD’s large cavity. LptD’s transmembrane β-barrel is exceptionally large for a bacterial protein, consisting of 26 individual β-strands and spanning 50 Å. This large cavity structure is needed to export the diverse, flexible OS moieties of LPS, but also produces a large gap in the membrane. The crystal structure of LptD/E shows that LptE is positioned between the extracellular loops of the LptD β-barrel, blocking part of the extracellular opening [30]. Grabowicz et al. identified an LptE mutant that caused an increase in membrane permeability without affecting LptD assembly or LPS export [34]. No individual change produced the same high-permeability phenotype, but the four most N-terminal mutations all affect residues near the extracellular end of LptE.

LptE has been shown to bind and break up surface-bound aggregates of LPS in vitro [30,35]. It is difficult to disentangle LptE’s in vivo interactions with LPS from its other established roles in LptD assembly and plugging, as disrupting either process also increases membrane permeability and hinders LPS transport. However, Malojcic et al. found that mutating the positively-charged residues (R91D, K136D) in a loop homologous to the LPS binding sites in other LPS-binding proteins disrupted binding in vitro and increased in vivo membrane permeability without affecting LptD assembly [30]. Based on this and the observation that LptE will break up surface-bound LPS aggregates, they proposed that LptE facilitates the final transfer of LPS to the outer leaflet by preventing their aggregation at the inner leaflet. Interestingly, one of Malojcic’s rationally-designed mutations, K136D, is on the same locus as the K136W mutation in Grabowicz’s randomly-generated plugging-deficient LptE14 mutant. This implies a degree of functional overlap between the structural components of LptE.

The LptD/E complex’s location at the extracellular surface makes it a more promising antibiotic target than the other Lpt subunits. Antibiotic compounds do not need to permeate the LPS barrier to reach it, and disrupting plugging of the LptD β-barrel could complement other antibiotics by increasing the permeability of the OM. The compound murepavadin and peptide mimetics have been shown to prevent LPS barrier biogenesis in *Pseudomonas aeruginosa* through direct interactions with LptD [36,37]. Crosslinking experiments have demonstrated that the peptide mimetic interacts with an extended region of the β-jellyroll domain found in the pseudomonads [38]. Andolina et al. suggested that this blocks the Lipid A channel, but other mechanisms influencing LptD assembly or LptA binding should not be discounted. Further structural characterization of the *P. aeruginosa* LptD periplasmic domain, which is currently unsolved, could elucidate this mechanism and provide clues for the rational development for more broad-spectrum antibiotics. LptE, meanwhile, may be less promising as a target as at least one species of bacteria, *Neisseria meningitidis*, can survive without LptE [39].

## 8. Summary

New structures and functional analyses of the Lpt system have made it possible to begin identifying the specific subunit-to-subunit interactions that make the continuous transport of LPS from the cytoplasm to the exterior of the outer membrane possible. The potentially novel type-III ABC mechanism employed by the LptB_2_FG subcomplex raises new questions about the mechanisms of LPS extraction and the ambiguous role of LptC. Determining how the Lpt system responds to shifts in the periplasmic space may contribute to a broader understanding of how Gram-negative bacteria adapt to environmental stresses affecting the cellular envelope. The LptD/E OM complex has proven a promising target for new antibiotics, which could be further refined through rational design informed by the recent structural characterization of its export mechanism.

## Abbreviations

ABC	ATP-binding cassette
EPR	electron paramagnetic resonance
IM	inner membrane
LPS	lipopolysaccharide
Lpt	lipopolysaccharide transport
NBD	nucleotide binding domain
OM	outer membrane
OS	oligosaccharide
TMD	transmembrane domain
